# Scaling-up Health-Arts Programmes: the largest study in the world bringing arts-based mental health interventions into a national health service

**DOI:** 10.1192/bjb.2020.122

**Published:** 2021-02

**Authors:** Carolina Estevao, Daisy Fancourt, Paola Dazzan, K. Ray Chaudhuri, Nick Sevdalis, Anthony Woods, Nikki Crane, Rebecca Bind, Kristi Sawyer, Lavinia Rebecchini, Katie Hazelgrove, Manonmani Manoharan, Alexandra Burton, Hannah Dye, Tim Osborn, Lucinda Jarrett, Nick Ward, Fiona Jones, Aleksandra Podlewska, Isabella Premoli, Fleur Derbyshire-Fox, Alison Hartley, Tayana Soukup, Rachel Davis, Ioannis Bakolis, Andy Healey, Carmine M. Pariante

**Affiliations:** 1 Department of Psychological Medicine, Institute of Psychiatry, Psychology and Neuroscience, King's College London, UK; 2 Department of Behavioural Science and Health, Institute of Epidemiology and Health Care, University College London, UK; 3 Department of Basic and Clinical Neuroscience, Institute of Psychiatry, Psychology and Neuroscience, King's College London, UK; 4 Parkinson Foundation International Centre of Excellence, King’s College Hospital and Kings College London, UK; 5 Centre of Implementation Science, Health Service and Population Research Department, Institute of Psychiatry, Psychology & Neuroscience, King's College London, UK; 6 King's Cultural Community, King's College London, UK; 7 South London and Maudsley NHS Foundation Trust, Maudsley Hospital, UK; 8 Breathe Arts Health Research, The Clarence Centre, London, UK; 9 Rosetta Life, Chipping Norton, UK; 10 Department of Clinical and Motor Neuroscience, UCL Queen Square Institute of Neurology, London, UK; 11 The National Hospital for Neurology and Neurosurgery, London, UK; 12 Faculty of Health, Social Care and Education, Centre for Health and Social Care Research, Kingston University and St George's, University of London, UK; 13 English National Ballet, London, UK; 14 Health Services and Population Research Department, Institute of Psychiatry, Psychology & Neuroscience, King's College London, UK; 15 Department of Biostatistics and Health Informatics, Institute of Psychiatry, Psychology & Neuroscience, London, UK

**Keywords:** Perinatal psychiatry, randomised controlled trial, neuroimmunology, patients, psychosocial interventions

## Abstract

The Scaling-up Health-Arts Programme: Implementation and Effectiveness Research (SHAPER) project is the world's largest hybrid study on the impact of the arts on mental health embedded into a national healthcare system. This programme, funded by the Wellcome Trust, aims to study the impact and the scalability of the arts as an intervention for mental health. The programme will be delivered by a team of clinicians, research scientists, charities, artists, patients and healthcare professionals in the UK's National Health Service (NHS) and the community, spanning academia, the NHS and the charity sector. SHAPER consists of three studies – Melodies for Mums, Dance for Parkinson's, and Stroke Odysseys – which will recruit over 800 participants, deliver the interventions and draw conclusions on their clinical impact, implementation effectiveness and cost-effectiveness. We hope that this work will inspire organisations and commissioners in the NHS and around the world to expand the remit of social prescribing to include evidence-based arts interventions.

The field of arts and mental health is a constantly and rapidly expanding area of research. We now have numerous publications, in the UK and globally, suggesting a strong link between arts-based interventions and improvement in mental and physical health outcomes.^1–9^ This invaluable body of research has shown the clinical effectiveness of the arts for the treatment of an array of mental and physical health problems, as diverse as anxiety, depression, cancer, cerebral palsy and stroke, among others. However, such research has yet to offer solutions that are readily scalable, implementable and cost-effective, and that can be employed in primary and secondary care settings in the National Health Service (NHS) or equivalent health services in other countries. Furthermore, these art interventions face limitations, including the lack of a continuous stream of funding, limited partnerships with commissioners and funders, insufficient clinical evidence and difficulties in their implementation in existing clinical pathways.

## The SHAPER project

The Scaling-up Health-Arts Programmes: Implementation and Effectiveness Research (SHAPER) project aims to start addressing the above gaps in the evidence base. SHAPER is a multidisciplinary programme funded by the Wellcome Trust, and is being run by the Institute of Psychiatry, Psychology & Neuroscience at King's College London and by the Department for Behavioural Science and Health and the Institute of Mental Health at University College London. The project aims to scale up three existing community arts interventions: Melodies for Mums (for women with postnatal depression), Dance for Parkinson's, and Stroke Odysseys. These three interventions have been developed and piloted on a small scale, offering promising results within their rehabilitation scopes.^10–14^

SHAPER was designed following an intensive 6-month scoping process involving stakeholders at different levels, including artists, clinicians, patients, researchers and commissioners. The aims were to create a programme that meets specific needs in the healthcare sector, to scale up interventions that already had promising efficacy pilot data and to involve high-quality arts interventions led by experienced partners (e.g. Breathe Arts Health Research, the English National Ballet and Stroke Odysseys). It has the ambition to be inclusive of the larger patient population (including those not already engaged in the arts) and, importantly, to be scalable and commissionable by the healthcare sector.

The ambition of the programme is thus to embed arts interventions within a large academic health science centre, King's Health Partners (KHP), establishing their delivery in the medium- to long-term future. As an academic health sciences centre, KHP's academic and clinical partners (King's College London, Guy's and St Thomas’ NHS Foundation Trust, King's College Hospital NHS Foundation Trust, and South London and Maudsley NHS Foundation Trust) bring together research, education and clinical practice for the benefit of the patients. SHAPER's objective is to study the three arts interventions embedded within existing clinical pathways in order to scale them up to reach larger numbers of people across KHP and the community, examine possible mechanisms of efficacy and provide implementation evidence.

The SHAPER programme intends, ultimately, to enable clinical commissioning groups (CCGs; i.e. the ‘payers’ in the NHS) to commission the three interventions, so that they can continue to be delivered in the future. To attain the aims mentioned above, three levels of effectiveness must be assessed: (a) clinical effectiveness, considering the real-world impact on health outcomes and whether these are meaningful to clinical practice; (b) implementation effectiveness, in terms of uptake, suitability, acceptability and feasibility of the interventions; and (c) cost-effectiveness, to develop strong business plans for commissioners. Since the interventions selected are at different developmental stages, an additional ambition of SHAPER is that the implementation science methodologies developed in this programme will allow the creation and evaluation of an implementation model that could be used across future arts interventions, tailored to different stages of development and delivery.

The adopted research, a three-pronged hybrid type II effectiveness-implementation evaluation, is the gold standard of modern implementation science that blends components of clinical effectiveness and implementation research.^15^ This strategy will allow the research team to simultaneously test the clinical intervention and the implementation strategy, and our team has used it successfully in previous scale-up research at King's College London.^15,16^

The programme is in the process of obtaining ethics committee approvals. Consent will be sought from all research participants and stakeholders involved in these studies.

## Cross-cutting implementation and health economics evaluation

An innovative aspect of the SHAPER programme is that, in addition to the clinical effectiveness of the interventions, a systematic approach will be taken to simultaneously evaluate their implementation effectiveness and cost-effectiveness. Implementation effectiveness refers to the uptake, suitability, acceptability and feasibility of the interventions. This will help us to identify not just ‘if’, but also ‘why’ and ‘how’ the interventions work and for whom, and will support our understanding of how they can be successfully delivered within clinical pathways. This approach of simultaneously assessing clinical, implementation and cost-effectiveness of an intervention is supported by the recently emerged ‘hybrid’ research designs – which offer a framework for these different elements of effectiveness to be assessed in parallel.^15^ Overall, the SHAPER programme is conceptualised as a hybrid type II design, in which the clinical and implementation effectiveness are given equal weight in the design of the evaluation across the three focal interventions.

The evaluation of how the interventions are implemented within clinical pathways will further capture barriers and drivers of implementation as well as unintended consequences for patients and providers. Existing implementation theory and direct work with a wide group of stakeholders of the interventions (including patients, arts and clinical providers) will inform the implementation evaluation throughout the programme.^17,18^ Along similar lines, the cost-effectiveness evaluation will cover health economic evaluations of the implementation and delivery costs and associated savings, service utilisation and related analyses. Implementation and cost data will be captured through a mixed-methods approach comprising a variety of qualitative and quantitative data collection techniques, including structured interviews and psychometrically established measurement scales.^19^

In bringing these three studies together, the SHAPER programme aims to bridge the gap between small-scale arts interventions and their large-scale implementation into pathways within the NHS for improved physical and mental health in people with postnatal depression, Parkinson's disease and stroke.

Here, we offer an overview of the three interventions and of the implementation and cost-effectiveness evaluation adopted within SHAPER.

## Melodies for Mums (arts partner: Breathe Arts Health Research)

Postnatal depression affects at least 12.9% of new mothers, with symptoms including fatigue, anhedonia, insomnia and irritability.^20,21^ However, challenges surround the fact that there is still no complete treatment solution: although pharmacological treatments have had positive results, these are hampered by low uptake and adherence among mothers.^22–24^ Psychotherapy has also produced mixed results, as well as similar challenges regarding low uptake or delayed treatment.^21,25–27^ However, many mothers engage in community group activities with their babies, such as attending mother–infant play groups. These activities have been identified as ways of relaxing mothers, providing good sources of social interaction, decreasing the monotony of each day and providing a sense of personal fulfilment.^28^

Moreover, there is a growing body of evidence demonstrating the effects of community group singing on mental health.^29,30^ Singing to new babies is practised in cultures around the world, and research has demonstrated valuable benefits, such as improving mother–infant interaction and reducing distress in babies.^31–33^ Listening to music during pregnancy is also associated with higher levels of well-being and reduced symptoms of postnatal depression in the first 3 months post-birth, while daily singing to babies is associated with fewer symptoms of postnatal depression and higher levels of well-being, self-esteem and perceived mother–infant bond.^10^ Consequently, there is a strong theoretical background indicating that singing could support mothers with postnatal depression.

**Fig. 1:**
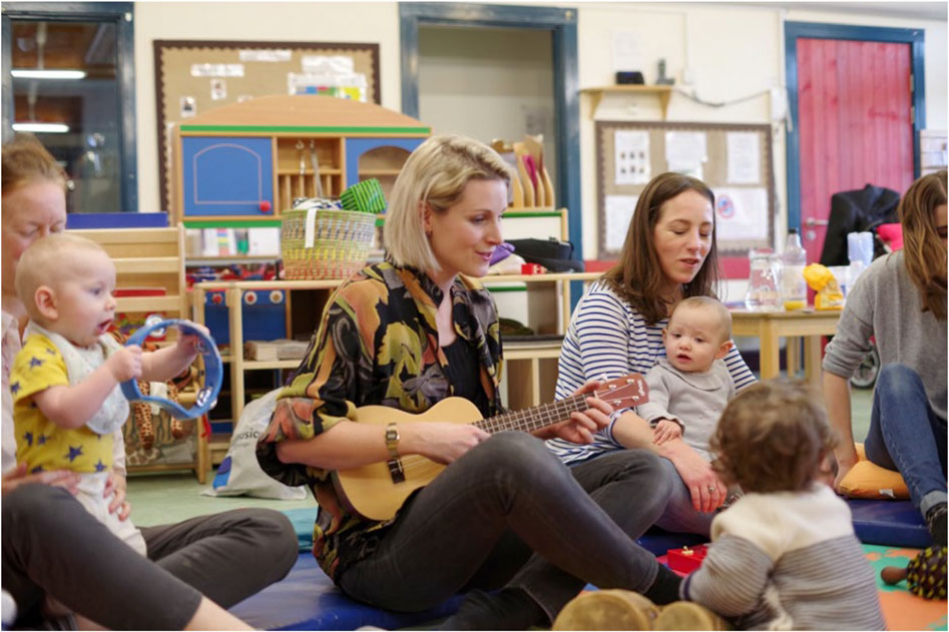
Melodies for Mums session delivered by Breathe Arts Health Research. Image credit: Richard Eaton.

Breathe Arts Health Research's Melodies for Mums offers free, community-based singing sessions to women with symptoms of postnatal depression in London boroughs (Fig. 1). A previous study led by researchers in the SHAPER team has shown that this intervention, already implemented in some London boroughs, results in faster improvements in symptoms when compared with usual care.^11^ Specifically, the study recruited 134 women with symptoms of postnatal depression and found that, in women with moderate to severe depression, there was significantly faster improvement in symptoms in the singing group than in the group play workshops for mothers and babies.

The planned two-arm randomised clinical trial (SHAPER-PND) aims to establish effectiveness in a larger sample (400 participants) and to analyse the factors affecting economic and implementation potentials for this intervention. Participants will be assigned to either a 10-week singing intervention or a 10-week active waiting-list control group, where they will be encouraged to attend community mother–baby activities. Singing sessions will be delivered in children's or community centres and each group will have 8–12 mothers and their babies. Mothers and their babies will be invited to sit in a circle and learn songs from all over the world, from vocal ‘motherese’ style noises to lullabies and more complex songs. They will be invited to hug or stroke their babies while singing and to add simple musical instruments (maracas, drums, hand chimes and others) to increase mother–baby interactions. Mothers will also be invited to develop their own songs about motherhood and their babies, creating a shared experience with other participants, thereby increasing their sense of inclusion.

A package of demographic, mental health, biological and social measures will be collected from mothers and babies at set time points throughout the intervention, and up to 36 weeks post-randomisation. In addition to the clinical effectiveness outcome (an improvement in depressive symptom score according to the Edinburgh Postnatal Depression Scale), we will put equal weight on the implementation science and economic data assessments. For biological outcomes, we will collect saliva and hair samples to assess stress and hormonal markers, including cortisol, oxytocin and cytokines. We will also conduct qualitative interviews with a subgroup of mothers who self-report particular risk factors for postnatal depression, to explore how singing interacts with specific contexts.

## Dance for Parkinson's (arts partner: English National Ballet)

Parkinson's disease is a chronic neurodegenerative condition affecting over 145 000 people in the UK alone, with a prevalence expected to rise by around 18% between 2018 and 2025, to over 168 000, and to double by 2065.^34^ Parkinson's disease is a complex disorder characterised by a range of motor symptoms, including slowness of movement (bradykinesia), tremor and gait impairment, and non-motor symptoms such as anxiety, depression, sleep dysfunction, autonomic problems, mood disturbances and cognitive decline, with a profound negative effect on quality of life.^35^ Although there are no treatments that can affect the progression of this condition, evidence is emerging that physical activity and certain types of exercise, including a range of dance-based exercise, can improve motor symptoms, functional mobility and stability, and result in some cognitive improvements, reduced pain, depression and anxiety, decreased social isolation and improved quality of life.^36–38^

**Fig. 2:**
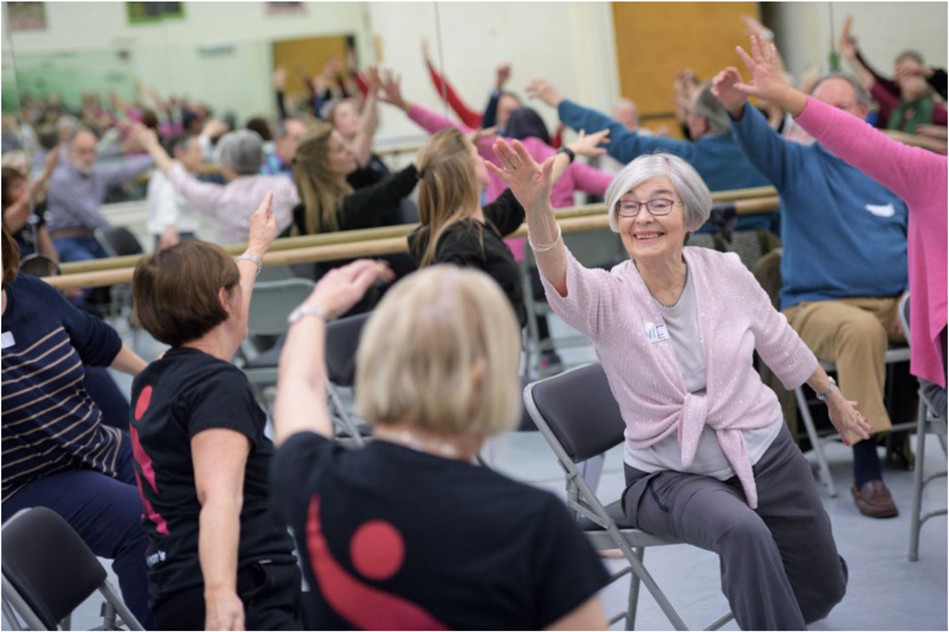
Dance for Parkinson's session delivered by the English National Ballet. Image credit: Laurent Liotardo.

Dance for Parkinson's is an existing programme delivered by the English National Ballet (ENB) for people with Parkinson's disease (Fig. 2) across multiple venues in the UK, including London, Ipswich, Cardiff, Liverpool and Oxford. The sessions are being delivered by ENB-trained dance artists and currently host people with Parkinson's and carers. These sessions are popular and lend themselves to the requirement of a large-scale randomised study so as to provide robust evidence of dance being accepted as a potential therapeutic option in the pathway of care for Parkinson's disease.

As part of the SHAPER project, the study will be supported by the Wellcome Trust and will take place at the internationally renowned Parkinson's Foundation Centre of Excellence at King's College Hospital and King's College London. It will be a two-arm randomised controlled trial (SHAPER-PD-Ballet) to investigate the clinical efficacy of the intervention in a larger sample (160 participants), graded by the severity of their Parkinson's (mild, moderate and severe). Participants will be randomly allocated to receive 12 weekly ballet classes delivered by a team of ENB-trained dance artists and musicians, lasting approximately 75 min and incorporating live music, dance, rhythm and voice. A comparator group will continue on conventional treatment regimes. Participants will be followed up for up to 6 months post-intervention, and those allocated to the comparator group will be offered participation in the ballet sessions at the end of the project.

The clinical aspect of the study will, for the first time, use a range of clinically validated outcome measures, including the comprehensive version of the Non-Motor Symptom Scale developed at King's College Hospital. Secondary outcome measures include assessments of both motor and non-motor symptoms, such as cognitive decline, mood, sleep and pain. Additionally, wearable sensors will provide an objective measure of the Parkinson's signs as well as mobility and balance. Assessment quality will be checked by a ‘masked/blinded’ rater.

A unique aspect will be that all participants will be offered participation in a substudy of electrodiagnostic measures, which will employ transcranial magnetic stimulation coupled with electroencephalography and electromyography to investigate the effects of the intervention on neural networks and brain activity.

Implementation and economic data will be collected to assess acceptability, appropriateness and feasibility of the intervention on a large scale and its potential to be adopted and sustained as a cost-effective and beneficial adjuvant therapy. All measurements will be conducted at baseline (before the start of the intervention), immediately post-intervention between 3 and 6 months post-intervention to explore the acute and chronic benefits.

To our knowledge, this is the first randomised controlled trial investigating the effects of ballet dancing on people with neurological disorders.

## Stroke Odysseys (arts partner: Rosetta Life)

Stroke is a leading cause of disability in the UK and worldwide, and approximately two-thirds of stroke survivors leave hospital with disability.^39^ There are over 1.2 million stroke survivors in the UK, projected to exceed 2 million by 2035. Stroke costs the UK an estimated £25.6 million annually.^40^ Recent data from the Sentinel Stroke National Audit Programme shows that nearly 40% of patients between August and November 2017 left hospital with moderate to severe disability (modified Rankin scale, 3–5).^41^ Indeed, the transition from hospital to home after a life-changing event such as a stroke is extremely difficult both for the individual concerned and for their family, friends and caregivers. Fragmentation of health services often means that information provision relating to discharge is poor, which may also contribute to delays in discharge from hospital.

**Fig. 3:**
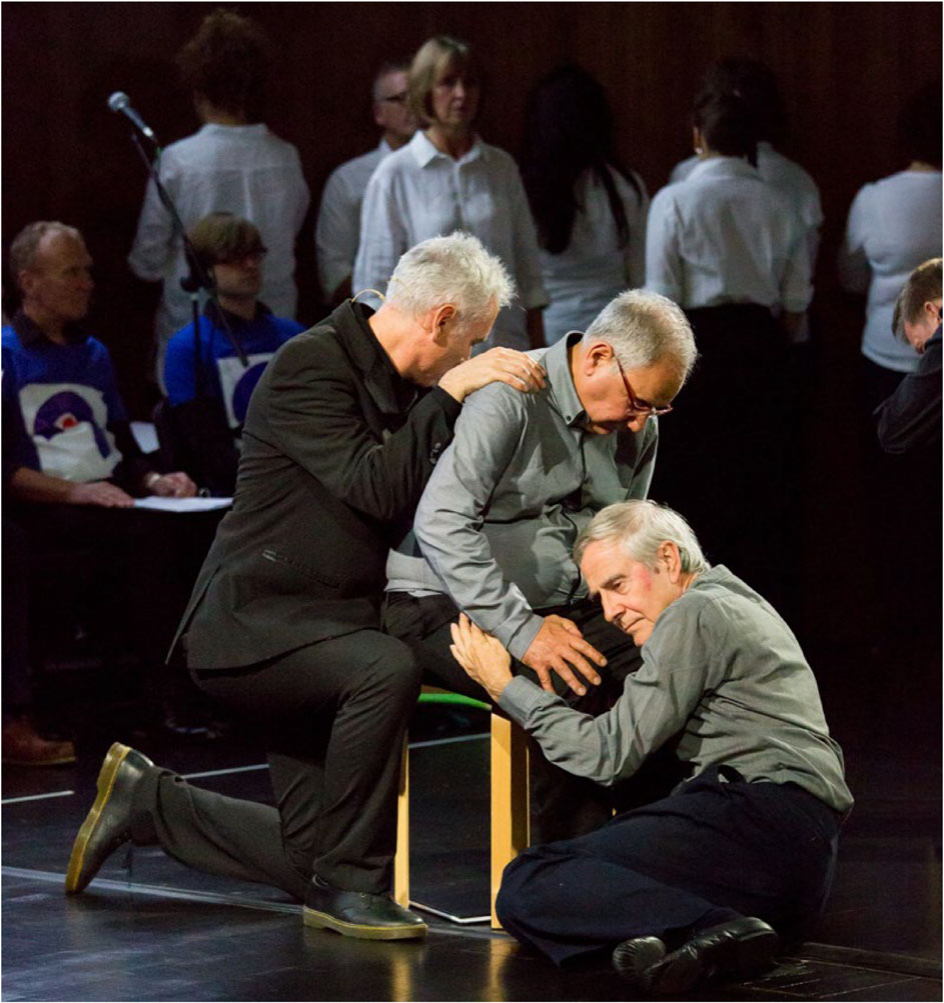
Stroke Odysseys tour performance. Image credit: Rosetta Life.

Stroke Odysseys, a post-stroke performance arts intervention, has been co-designed by artists and developed by the organisation Rosetta Life in a unique partnership with south London stroke communities. It is an intervention using performance arts to support recovery, agency and well-being in stroke survivors (Fig. 3). The intervention was initially developed and funded by King's and Guy's and St Thomas’ Charity and has been delivered in four London boroughs.^13^

Stroke Odysseys has three stages – clinical intervention, community intervention and stroke ambassadors – all of which will be replicated in this study. During the clinical intervention, while the patient is in hospital, the sessions will run for 60 min for groups of 6–8 patients in neuro-rehabilitation wards. These sessions will be led by a trained movement artist and a singer, and will involve movement, performance exercises, vocal warm-ups and singing. Dance practices will be rooted in improvisation, somatic dance theory and carnival/folk dance. Then, in the community stage, patients will be invited to perform their own stories in a 12-week performance intervention, working with performance arts towards creating a new perception of their own identity post-stroke. The performance is based on skills acquired in movement, music, song and spoken word, which has not only demonstrated benefits on perception of disability and cognition, but also aims to manage the anxiety and depression that affects one-third of stroke survivors.^42,43^ Finally, in the third stage, participants who complete the community intervention will be invited for training to become advocates for life after stroke: ‘stroke ambassadors’. Stroke ambassadors support the running of the programme in hospitals, assisting artists, recruiting participants and performing. Stroke ambassadors also speak at conferences and at regional stroke association groups and are members of an integrated performance company that create performance works to advocate for life after stroke. The study aims to recruit 75 new ambassadors. The main aim of the study is to evaluate the implementation, impact and experiences of a community-based performance arts programme (Stroke Odysseys for stroke survivors) using mixed methods (interviews, observations and surveys) prior to and after each programme stage, and carry out non-participant observations during the workshops. A series of implementation measures will be used as well as clinical outcome measures, including the Oxford Participation and Activities Questionnaire, a patient-reported outcome measure that assesses patients experiencing a range of health conditions. In addition, a health economic evaluation will be performed to cost the resources used in implementing the programme, and to evaluate wider service utilisation and associated costs before and after participants complete the programme and any changes in their quality of life profile.

## Conclusions

Our ambition is that the SHAPER programme will not only provide conclusive clinical and mechanistic evidence on the three studies described above, but also offer an invaluable resource to shape the future of arts interventions within the realm of rehabilitation for a range of other mental and physical health conditions.

SHAPER also presents as a unique opportunity to build a strong evidence base on the clinical effectiveness, implementation and mechanisms of arts interventions. Such a knowledge base will bring arts interventions into mainstream psychiatric care and put them on an equal footing with other pharmacological and psychosocial approaches.

## About the authors

**Carolina Estevao** is a Postdoctoral Research Associate and Clinical Project Manager in the Department of Psychological Medicine, Institute of Psychiatry, Psychology & Neuroscience (IoPPN), London, UK. **Daisy Fancourt** is an Associate Professor of Psychobiology and Epidemiology and Wellcome Research Fellow in the Psychobiology Group in the Department of Behavioural Science and Health, University College London (UCL), London, UK. **Paola Dazzan** is the Professor of Neurobiology of Psychosis in the Department of Psychological Medicine, IoPPN, London, UK. **Ray Chaudhuri** is the Professor of Neurology/Movement Disorders in the Maurice Wohl Clinical Neuroscience Institute, King's College London and Medical Director of the Parkinson Foundation International Centre of Excellence, King’s College London, UK. **Nick Sevdalis** is a Professor of Implementation Science & Patient Safety in the Centre for Implementation Science, Health Service and Population Research Department, IoPPN, London, UK. **Anthony Woods** is the SHAPER Programme Manager in the Department of Psychological Medicine, IoPPN, King's College London, UK. **Nikki Crane** is the SHAPER Programme Lead in the Culture Team, King's College London, UK. **Rebecca Bind** is a Postdoctoral Research Associate and Clinical Trial Manager in the Department of Psychological Medicine, IoPPN, London, UK. **Kristi Sawyer** is a Doctoral Student in the Department of Psychological Medicine, IoPPN, London, UK. **Lavinia Rebecchini** is a Research Assistant in the Department of Psychological Medicine, IoPPN, London, UK. **Katie Hazelgrove** is a Postdoctoral Research Associate in the Department of Psychological Medicine, IoPPN, London, UK. **Manonmani Manoharan** is a Consultant Psychiatrist with the South London and Maudsley NHS Foundation Trust, UK. **Alexandra Burton** is a Senior Research Fellow in the Department of Behavioural Science and Health, UCL, UK. **Hannah Dye** is the Head of Programmes with Breathe Arts Health Research, The Clarence Centre, London, UK. **Tim Osborn** is a Project Manager with Breathe Arts Health Research, The Clarence Centre, London, UK. **Lucinda Jarrett** is the Creative Director in the Rosetta Life Head Office, Chipping Norton, UK. **Nick Ward** is the Professor of Clinical Neurology and Neurorehabilitation in the Department of Clinical and Motor Neuroscience in the Clinical Neuroscience Centre, London, UK and Honorary Consultant Neurologist at the National Hospital for Neurology and Neurosurgery, Queen Square, London, UK. **Fiona Jones** is the Professor of Rehabilitation Research in the Faculty of Health, Social Care and Education, Kingston University and St George's, University of London, UK. **Aleksandra Podlewska** is a Doctoral Student in the Maurice Wohl Clinical Neuroscience Institute, King's College London, UK. **Isabella Premoli** is a Postdoctoral Researcher in the Maurice Wohl Clinical Neuroscience Institute, King's College London, UK. **Fleur Derbyshire-Fox** is the Director of Engagement with the English National Ballet, London, UK. **Alison Hartley** is the Head of Creative Programmes with English National Ballet, London, UK. **Tayana Soukup** is a Postdoctoral Researcher in the Centre for Implementation Science, Health Service and Population Research Department, IoPPN, London, UK. **Rachel Davis** is a Senior Research Fellow in the Centre for Implementation Science, Health Service and Population Research Department, IoPPN, London, UK. **Ioannis Bakolis** is the Senior Lecturer in Biostatistics and Epidemiology in the Centre for Implementation Science, Health Service and Population Research Department, IoPPN. **Andy Healey** is a Senior Health Economist in the King's Health Economics, Health Service and Population Research Department, IoPPN, London, UK. **Carmine M. Pariante** is the Professor of Biological Psychiatry in the Department of Psychological Medicine, IoPPN, London, UK.

## Supplementary material

For supplementary material accompanying this paper visit https://doi.org/10.1192/bjb.2020.122. click here to view supplementary material
